# Two-Dimensional Graphitic Carbon Nitride (g-C_3_N_4_) Nanosheets and Their Derivatives for Diagnosis and Detection Applications

**DOI:** 10.3390/jfb13040204

**Published:** 2022-10-26

**Authors:** Mehrab Pourmadadi, Maryam Rajabzadeh-Khosroshahi, Fatemeh Saeidi Tabar, Narges Ajalli, Amirmasoud Samadi, Mahsa Yazdani, Fatemeh Yazdian, Abbas Rahdar, Ana M. Díez-Pascual

**Affiliations:** 1 School of Chemical Engineering, College of Engineering, University of Tehran, Tehran 14179-35840, Iran; 2 Department of Chemical and Biomolecular Engineering, 6000 Interdisciplinary Science & Engineering Building (ISEB), Irvine, CA 92617, USA; 3 Department of Biomedical Engineering, State University of New York at Buffalo, Buffalo, NY 14260, USA; 4 Department of Life Science Engineering, Faculty of New Science and Technologies, University of Tehran, Tehran 14179-35840, Iran; 5 Department of Physics, Faculty of science, University of Zabol, Zabol 538-98615, Iran; 6 Universidad de Alcalá, Facultad de Ciencias, Departamento de Química Analítica, Química Física e Ingeniería Química, Ctra. Madrid-Barcelona, Km. 33.6, 28805 Alcalá de Henares, Madrid, Spain

**Keywords:** diagnosis, graphitic carbon nitride, biosensors, nanomaterials, antimicrobial activity biomedical applications

## Abstract

The early diagnosis of certain fatal diseases is vital for preventing severe consequences and contributes to a more effective treatment. Despite numerous conventional methods to realize this goal, employing nanobiosensors is a novel approach that provides a fast and precise detection. Recently, nanomaterials have been widely applied as biosensors with distinctive features. Graphite phase carbon nitride (g-C_3_N_4_) is a two-dimensional (2D) carbon-based nanostructure that has received attention in biosensing. Biocompatibility, biodegradability, semiconductivity, high photoluminescence yield, low-cost synthesis, easy production process, antimicrobial activity, and high stability are prominent properties that have rendered g-C_3_N_4_ a promising candidate to be used in electrochemical, optical, and other kinds of biosensors. This review presents the g-C_3_N_4_ unique features, synthesis methods, and g-C_3_N_4_-based nanomaterials. In addition, recent relevant studies on using g-C_3_N_4_ in biosensors in regard to improving treatment pathways are reviewed.

## 1. Introduction

The early detection of the biomarkers of the diseases plays a significant role in their treatment and control. It is essential to detect biomarkers associated with a disease early and with the high precision for diagnosis, treatment, and prognosis of fatal diseases, such as cancer, which causes a high mortality rate yearly, and neurodegenerative disorders [1,2,3,4]. There are some current conventional diagnostic methods, such as blood tests, imaging, and biopsies, which can be expensive and time-consuming with low sensitivity. Moreover, they require trained personnel, limiting their availability to low-income patients [3]. 

Today, biosensors are used for detection approaches, such as the high-resolution imaging, fast detection, and monitoring of diseases. Biosensors consist of three main components: recognition, signal transducer, and processor, designed to determine specific biomolecules [5]. These biomolecules can be macromolecules, such as nucleic acid and proteins, or small molecules, such as glucose. Various cancer biomarkers, such as BRCA1, BRCA2, CA 15-3, and CA 125 for breast cancer and PSA for prostate cancer, can be detected as well [6]. 

Nanotechnology has allowed advances in monitoring, diagnosis, prognosis, and proposing effective treatments [7,8,9,10,11,12,13,14,15,16]. In this sense, biosensors based on nanomaterials have accurate detection, efficient monitoring, and fast but reliable imaging [17,18]. The physicochemical properties of nanomaterials, such as photoemission, high specific surface leading to extra bioreceptor immobilization, as well as electrical and heat conductivities, make them perfect candidates for biosensor construction [19,20,21,22,23]. Graphene/graphene oxide, carbon quantum dots, gold nanoparticles, carbon nanotubes, porous carbon, and fullerene are nanostructures that have been investigated as the biosensing platforms studied over the years [24,25,26,27,28,29,30,31,32,33]. Carbon nanostructure-based sensors are utilized due to their potential to quench fluorescently-labeled probes [16,17,18,19,20,21]. Thus, developing a user-friendly and highly sensitive biosensor is essential. Graphitic carbon nitride (g-C_3_N_4_) nanosheet is another widely used carbon nanostructure to design biosensors [34,35,36,37,38,39]. g-C_3_N_4_ nanosheets have high fluorescence quantum yield, superior chemical and thermal stability, are easy to synthesize with low toxicity, and have a low price and high biocompatibility together with unique photoelectrochemical and electroluminescent characteristics [40,41]. Furthermore, the optical properties and conductivity of g-C_3_N_4_ have made it applicable in optical and electrochemical biosensing approaches. For instance, sulfur-doped graphitic carbon nanosheets (s-g-C_3_N_4_) as a dual (electrochemical and fluorescence) biosensing platform were used for the detection of cancer biomarkers even at very low concentrations (CA15-3) [42]. This review summarizes the properties and synthesis methods of graphitic carbon nitride nanosheets, which make them highly suitable candidates for the next generation of biosensors. 

## 2. g-C_3_N_4_-Based Materials: Properties

g-C_3_N_4_ is a polymeric nanosheet with a graphene-like structure consisting of sp^2^ bonded carbon and nitrogen atoms with abundant amino groups on its surface and suitable bandgap energy of 2.7 eV [43]. Thanks to the g-C_3_N_4_ electronic band structure with sp^2^ hybridization, it is considered a photon-harvesting semiconductor material that plays a critical role in detecting biomolecules by photoelectrochemical (PEC) biosensors [44]. Due to the presence of melamine in the π-conjugated nanosheets, g-C_3_N_4_ is fluorescent with high photoluminescence quantum yield with high and minor absorption at 365 nm and visible light region, respectively [45,46], which can be quenched by materials, such as metal ions, nitrobenzene derivate, or biomolecules, such as heparin and sialic acid, which allow its use as a fluorescent probe biosensor [47] with high photostability and no obvious photobleaching under UV light excitation for 10 h [48]. Furthermore, the g-C_3_N_4_ ability to convert light and electricity makes it a suitable option for electrochemiluminescence-based and photoelectrochemistry-based biosensing [39]. Various precursors have been proposed for g-C_3_N_4_ synthesis through thermal condensation. These compounds are rich in nitrogen and contain a tri-s-triazine ring structure, such as dicyandiamide, urea, cyanamide, or thiourea [49]. For instance, if cyanamide is selected as the precursor, thermal heating results in dicyanamide, melamine, melem, and g-C_3_N, respectively. 

**Figure 1:**
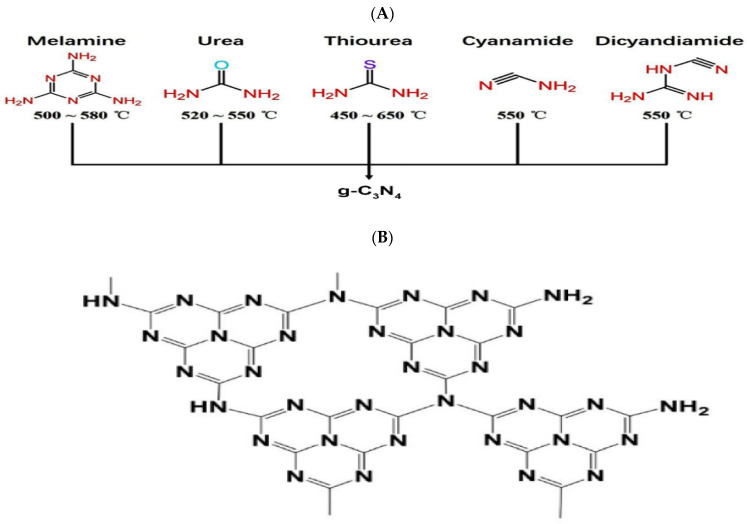
(**A**) Various g-C_3_N_4_ precursors and the corresponding temperatures for their thermal condensation into g-C_3_N_4_-, adapted from reference [50] under the terms and conditions of the Creative Commons Attribution (CC BY) license. (**B**) g-C_3_N_4_ structure, adapted from reference [51] under the terms and conditions of the Creative Commons Attribution (CC BY) license.

The molecular structures of the g-C_3_N_4_ precursors and the corresponding temperatures for their thermal condensation are depicted in Figure 1.

In addition, g-C_3_N_4_ has been reported to display antimicrobial activity. A number of parameters, including the g-C_3_N4 band gap, intermediate defect states, dispersed surface area, absorbance in suspension, and charge separation influence its photocatalytic bacterial inactivation [39]. Thus, the modification of this properties influences the production of reactive oxygen species, hence the antibacterial activity. The bactericidal rates of more than 99% have been successfully achieved for eight kinds of foodborne pathogenic bacteria with 8 h incubation in the dark. Cell rupture caused by direct mechanical contact between g-C_3_N_4_ and cell membranes has been observed. Molecular dynamics simulations further indicated that the presence of large defects in g-C_3_N_4_ enhanced the electrostatic attraction between inherent pores and lipid heads, resulting in enhanced antibacterial activity.

The thermal and chemical stability of biosensors is crucial for long shelf lives. g-C_3_N_4_ nanosheets show high thermal stability in the air (up to 600 °C) thanks to the graphitic graphene-like structure with sp^2^ bonds between carbon and nitrogen, providing high chemical stability [52]. g-C_3_N_4_ has low cytotoxicity and good biocompatibility due to its metal-free structure. Moreover, it has a low production cost, a simple synthesis process, a large specific surface area, easy functionalization, and increased penetration coefficient, allowing the efficient immobilization of molecules in the matrix for biosensing [53]. As g-C_3_N_4_ materials are increasingly used in biomedicine, improving their biocompatibility and biodegradability properties is a necessity. Therefore, modifications are applied to enhance the biocompatibility, biodegradability, and further development of g-C_3_N_4_ materials. For instance, Kang et al. showed that successfully inserting abundant disulfide bonds into g-C_3_N_4_ endowed more biodegradability and biocompatibility, boosting its application in biomedical fields [54]. In another study that was recently conducted for glucose detection in diabetic patients, the addition of metal co-catalysts (Fe(III), Cu(II)) to the structure via adsorption noticeably enhanced the sensitivity compared to the pristine g-C_3_N_4_ [55]. Thanks to its easy functionalization, g-C_3_N_4_ can be adapted to various targets with high sensitivity. For instance, a platform based on proton-functionalized ultrathin g-C_3_N_4_ nanosheets with a positive charge has been developed for heparin (as a biomolecule with a high negative charge) detection in human serum [56].

## 3. g-C_3_N_4_-Based Materials: Synthesis Methods 

### 3.1. Synthesis of g-C_3_N_4_ Nanosheets

The classification of the synthesis methods based on the synthesis procedure can be divided into bottom-up and top-down categories. The “bottom-up” approach generally applies small-sized particles to assemble complex structures. However, the “top-down” procedure is based on splitting large-sized and thick bulks into small particles and thin nanosheets [57,58]. The bottom-up procedure includes ionic liquid, supramolecular pre-assembly, and hydrothermal methods [58]. In the bottom-up approach, g-C_3_N_4_ sheets are synthesized on a large scale via thermal polymerization (pyrolysis) or the carbonization of small organic compounds (that contain hydroxyl, carboxyl, carbonyl, and primary amine functional groups) [59], such as melamine, cyanimide, Dicyanamide, or urea [60]. Dante et al. obtained g-C_3_N_4_ from the pyrolysis of melamine cyanurate at 650 °C for 50 min (in the crucible with atmosphere condition), which was used for glucose sensing [55]. On the other hand, chemical exfoliation and ultrasonic exfoliation methods have been utilized for the top-down approach. Chemical exfoliation is more common for large-scale production due to its high efficiency and the easier tuning of the g-C_3_N_4_ structure [61]. For example, Hatamie et al. used g-C_3_N_4_ as a label-free fluoro-sensor to analyze the amount of metronidazole in biological fluids and drug samples. g-C_3_N_4_ ultrathin nanosheets were synthesized in bulk via the thermal polymerization method from melamine, possessing a highly π-conjugated structure at 600 °C. The exfoliation procedure was performed through ultrasonication in water media [62]. 

### 3.2. Synthesis of g-C_3_N_4_-Based Composites

g-C_3_N_4_ properties can be enhanced through its fabrication with other materials into composites. In the modification techniques, metal loading is critical for increasing the potential application of g-C_3_N_4_ biosensors due to outstanding electrochemical qualities. Metal/g-C_3_N_4_ composites are produced with solvothermal treatment, photo-deposition, precipitation, and thermal polymerization methods [63]. Generally, there are numerous ways to prepare g-C_3_N_4_-based nanocomposites. The simple pyrolysis method, solution (sonication) mixing, the hydrothermal method, the simple calcination method, the hydrolysis method, sol-gel, and microwave irradiation are some synthesis methods that have been applied in the formation of nanocomposites based on g-C_3_N_4_- and have been utilized for different applications [43]. The pyrolysis method is a common way to produce g-C_3_N_4_-based composites in diagnosis applications where the mixture of the precursor of g-C_3_N_4_ and the other components is calcinated in a crucible for a while with a specific heating rate and initial temperature to prepare the nanocomposite. Then, the product is cooled at 25 °C. For example, a sensitive electrochemical sensor for dopamine detection was fabricated by firstly preparing calcium stannate (CaSnO_3_) nanoparticles from CaCl_2_ and SnCl_2_.2H_2_O via the hydrothermal method, then CaSnO_3_-gC_3_N_4_ nanohybrid was produced through the pyrolysis of melamine, (NH_4_)_2_SO_4_, and CaSnO_3_ mixture at 550 °C in a crucible [64]. In another study for glucose detection, Cu(II)–Fe(III)-g-C_3_N_4_ was prepared through the sonication method (2 h sonication of a suspension of 416 mg of g-C_3_N_4_ in a 20 mL aqueous solution containing Cu(II) and Fe(III) ions), which led to the adsorption of ions on the g-C_3_N_4_ structure [55]. A highly selective glucose-sensing (in human blood) biosensor based on ultrathin g-C_3_N_4_ nanosheets doped with niobium (Nb) metal was synthesized by the pyrolysis method from urea [65]. A biosensor for 4-nitrophenol detection was developed by Vinoth et al. 4-nitrophenol is a very poisonous chemical compound released into the water during the production of some drugs, dyes, and leather, posing human health at high risk. So, for 4-nitrophenol monitoring, the biosensor based on BaSnO_3_-g-C_3_N_4_ nanostructure was synthesized by sonication method from prepared BaSnO_3_ and g-C_3_N_4_ [66].

## 4. g-C_3_N_4_-Based Biosensors

### 4.1. g-C_3_N_4_-Based Surface Plasmon Resonance (SPR) Biosensors

Surface plasmon resonance (SPR) sensing is a powerful probe of the interplays between protein–ligand, protein–DNA, protein–protein, and protein–membrane binding [67]. SPR biosensors are a very effective tool for measuring many biomarkers [68]. The main advantages of these biosensors are their fast response and ability to detect various analytes concurrently [69]. Moreover, among various new techniques available, SPR biosensors are the best optical biosensors for label-free, fast, and in situ diagnosis of molecules [40]. SPR is a physical optics phenomenon that can detect biomarkers because of the high sensitivity of surface plasmons to the dielectric medium [70]. In these biosensors, receptors are immobilized on the metal surface, interacting with the analytes and leading to dielectric alteration. This phenomenon affects the resonance condition of surface plasmons with specific surface plasmon waves (SPWs), allowing the transmission of photon’s energy to the surface plasmons at the resonance angle resulting in the decrease of the light reflectance and thus the SPR curve [71]. Based on the characteristic of light, the SPR biosensors can be categorized into angular, wavelength, or intensity-modulated systems [72,73,74]. The Kretschmann configuration is the most recent version of SPR based on attenuated total reflection [54]. At an angle, part of light energy is transmitted to the surface plasmon, and the reflectance can be shown in the angular scanning. 

The presence of adsorbed molecules on the biosensor surface varies the refractive index, and the SPR angle is changed accordingly [75]. 

Two-dimensional (2D) materials with large surface areas, such as g-C_3_N_4_, can act as the sensitive layers for SPR [40]. Duan et al. designed a surface plasmon resonance (SPR) biosensor based on a 2D nanocomposite of g-C_3_N_4_ nanosheets and molybdenum disulfide quantum dots (MoS_2_QDs), adorned with chitosan-stabilized Au nanoparticles (CS-AuNPs) to detect prostate specific antigen (PSA) selectively. In this work, the MoS_2_QDs easily aggregated and reduced the sensitivity, but as a support for MoS_2_QDs, the g-C_3_N_4_ nanosheets improved the biosensing performance for PSA detection. Additionally, the MoS_2_QDs@ g-C_3_N_4_@ CS-AuNPs-based SPR aptasensor showed a very low limit of detection (LOD), 0.77 ng·mL^−1^, with good linearity range at PSA concentrations in the range of 1.0–250 ng·mL^−1^ [40]. 

### 4.2. g-C_3_N_4_-Based Electrochemical Biosensors

Electrochemical biosensors have been recognized as powerful diagnostic tests over the past years thanks to their unique advantages, such as simplicity, high sensitivity, and accuracy [76]. Three vital components are necessary to develop electrochemical biosensors: (I) a bioreceptor to link with analyte, (II) an electrode, and (III) a read-out system [77]. An electrochemical sensor requires a working reference and an auxiliary electrode; the working electrode in the electrochemical biosensor acts as a transducer in the reaction between the bioreceptor and the analyte. It generates a biological signal which changes into an electronic signal and is processed with high sensitivity [78]. On the other hand, Ag/AgCl-based reference electrode is kept at the site of the reaction to maintain a particular potential. The auxiliary electrode links the electrolytic solution and must be conductive; thus, gold or platinum are suitable candidates [79]. Some electrochemical methods for marker detection include voltammetric techniques (cyclic, square wave, or stripping), impedimetric, and amperometry. Of these techniques, cyclic voltammetry (CV) is preferred [77].

**Figure 2:**
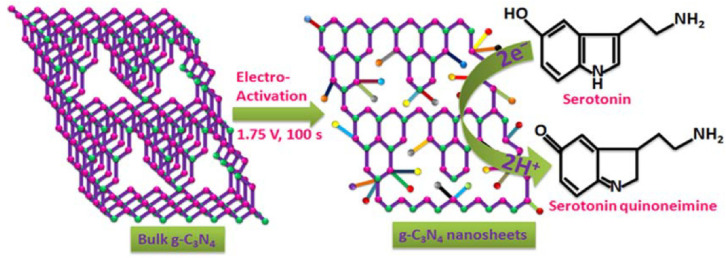
Activation of g-C_3_N_4_ on glassy carbon electrode and the redox reaction on the developed electrochemical biosensor for serotonin (5-HT)-. Adapted from reference [86] under the terms and conditions of the Creative Commons Attribution (CC BY) license.

In an electrochemical biosensor, an electrode is the main component for immobilizing electron motion and biomolecules [80]. Nanomaterials have piqued attention due to their unique electronic characteristics [81]. The carbon allotropes can be applied as electrodes due to their effective electron transfer rate and high active surface area. Additionally, carbon nanostructured materials are significant in research due to their unparalleled properties, such as chemical stability and good conductivity [82]. g-C_3_N_4_ is a polymeric semiconductor with a specific structure and high stability, making it a good nanocomposite for electrochemical biosensors [83]. g-C_3_N_4_ is known as the most thermal stable allotrope of carbon nitrides [84], which can be used in the diagnosis system based on its catalytic ability [85]. Due to the low electron conductivity of g-C_3_N_4_, it has been used with other materials to enhance its surface conductivity. The g-C_3_N_4_ derivatives can electrically connect to the redox center of biomolecules on the surface of the electrode. The electronic integration of the g-C_3_N_4_ with various carbon types notably increases the surface area and conductivity [85]. The chemical exfoliation of bulk g-C_3_N_4_ has been used to develop g-C_3_N_4_ nanosheets for the detection of neurotransmitters, such as dopamine (DA). Kathiresan et al. developed a glassy carbon electrode (GCE) doped with bulk g-C_3_N_4_. The electrochemical activation of bulk g-C_3_N_4_ was performed with a potential of 1.75 V in neutral pH conditions (pH 7.0). In the electrode oxidation reaction, the two-electron process is followed by the transfer of two protons, resulting in 5-HTquinoneimine. Figure 2 illustrates the redox reaction. Oxidation leads to the transfer of protons to form 5-HTquinoneimine and the reduction occurs in the quinone group on 5-HT quinoneimine [86].

Table 1 collects studies conducted on detecting various biomarkers using electrochemical biosensors.

### 4.3. g-C_3_N_4_-Based Photoelectrochemical (PEC) Biosensors

The photoelectrochemical (PEC) detection method is a hopeful technique for biological assays [135], which is also a low-cost approach to transforming chemical energy into electricity under a flash of light [136], and PEC biosensors have become prominent due to their capability of biomolecules diagnosis. This method has had much consideration because of its high sensitivity, simplicity, and fast response [137]. In the PEC diagnosis system, light is used as an excitation source [138], allowing for a high sensitivity with low background signals [136]. The PEC cell includes three main components: (a) a light-harvesting semiconductor, (b) a metal electrocatalyst, and (c) adequate electrolytes among the working electrode and auxiliary electrode to generate PEC signals using redox reaction. Upon illumination, the redox reactions lead to a signal between the working and the auxiliary electrodes [139]. 

PEC biosensors use wide bandgap semiconductors as photoactive materials [63], changing optical energy to electrical and chemical energy [140]. g-C_3_N_4_ is a responsive photocatalyst with a bandgap (2.7 eV) [141]. Additionally, one of the promising approaches is a photocatalytic reaction which can absorb visible light [82]. g-C_3_N_4_, as an inorganic polymeric semiconductor, possesses a graphite-like layer structure [142]. So, PEC biosensors show advantages over electrochemical and optical biosensors with high sensitivity and low cost. Hence research in the PEC biosensor for analyte detection has increased. Biomarkers detected using photoelectrochemical biosensors are summarized in Table 2. 

For instance, Li et al. developed a PEC biosensor based on coral-like g-C_3_N_4_ nanostructures to detect the metronidazole biomarker. Although metronidazole is a common antibacterial drug, it causes carcinogenic and genotoxic issues. Hence, the sensitive and facile detection of metronidazole’s residues in typical oral medicine samples is an effective approach in health care. According to the results, coral-like g-C_3_N_4_ nanostructures in the biosensor platform boosted the facility of signal amplification in the PEC sensing [168]. In the other study, Mao et al. applied the photosensitive CuO-g-C_3_N_4_ nanostructures as an efficient photocathode in the PEC sensing of aflatoxin B1 (as a food contaminator and class 1 carcinogen). The conjugation of CuO to g-C_3_N_4_ efficiently extended the optical absorption toward the visible region. The CuO-g-C_3_N_4_ nanocomposite enhanced the PEC signaling for the sensitive detection of aflatoxin B1 [145].

### 4.4. g-C_3_N_4_-Based Fluorescent Biosensors

**Figure 3:**
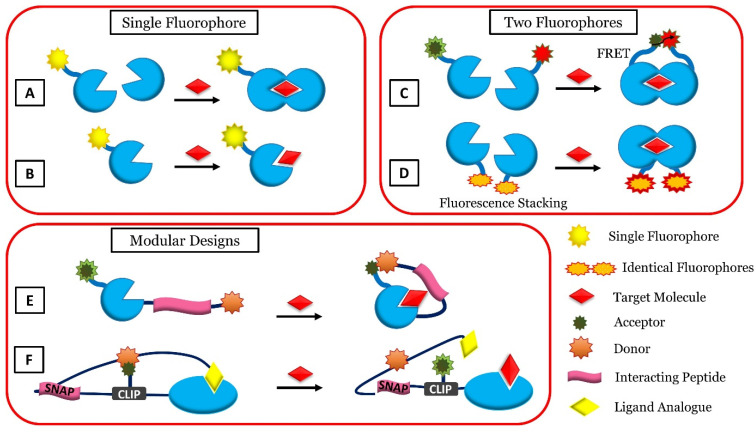
Different schemes of fluorescent reagent-less protein-based biosensors. Single-fluorophore-based biosensors: Change in conformation (**A**) or target interaction (**B**) changes the environment of fluorophore. Two-fluorophore-based biosensors: In between two different fluorophores, FRET is recorded (fluorescent proteins) (**C**), or by breaking the stack of two fluorescent dyes which are identical (**D**). Modular design-based biosensors: a part in the merged system with the recognition element can interact with either the target bound (**E**) or the target-free state (**F**) so that when the target binds, the signal is transduced, Reproduced from Ref. [185] under the terms and conditions of the Creative Commons Attribution (CC BY) license.

Fluorescent biosensors have been used in biological assays, owing to their high sensitivity, simple readout systems, lower response time, and visualization [175]. Fluorescent biosensors possess a specific ability to monitor biological cell targets [176,177]. Fluorescence spectroscopy has been widely applied to determine cancer and heavy metal ions [178,179]. Accordingly, the important advantages of this type of biosensor are that it is non-invasive, its capability to use fluorescence intensity, and its fluorescence lifetime. Additionally, using fluorescent nanomaterials, biomarker diagnosis can be highly selective and sensitive [180]. Fluorescent biosensors function by absorbing electromagnetic radiation, which is absorbed by fluorophores or fluorescently labeled molecules. Fluorescent biosensors can be divided into four types according to the signal-producing technique, including FRET (Forster Resonance Energy Transfer), FLIM (Fluorescence Lifetime Imaging), FI (Fluorescence Intensity and its change), and FCS (Fluorescence Correlation Spectroscopy) [181]. The fluorescence biosensors have a single signal for detection and can easily be disturbed by environmental and instrumental conditions [182]. In luminescence, light is produced by excitation without increasing the temperature. Fluorescence is a type of luminescence that occurs over a short period and is created by electromagnetic excitation [183]. Moreover, in fluorescence, the time interval between absorption and emission is short [184]. Figure 3 shows the various schemes of fluorescent reagent-less protein-based biosensors [185].

Nanomaterials have introduced an attractive method of developing low-cost and portable fluorescent devices [186]. In recent decades, a new group of 2D nanomaterials has attracted research attention. g-C_3_N_4_ nanosheets supply an iterating choice for bioimaging and bioprobes applications [187,188]. Additionally, the N-contain structure for the g-C_3_N_4_ nanosheet provides the potency for coordination with proton or metal ions [189]. The mentioned unique characteristics of g-C_3_N_4_ nanosheets make this useful for developing fluorescent biosensors or bioprobes. Table 3 shows some of the developed fluorescent biosensors for detecting different biomarkers.

Hatamie et al. applied g-C_3_N_4_ nanosheets to develop a label-free bioassay system for diagnosing metronidazole in biological fluids. The switch-off green fluorescence biosensor provided rapid sensing with a linear detection range from 0.01 to 0.10 μg mL^−1^ [62]. Dopamine is a neurotransmitter with substantial biological functions in neuroendocrine regulations, and its abnormal content in the human serum leads to Parkinson’s and Alzheimer’s disease. Lv et al. investigated the g-C_3_N_4_ nanofibers in the fluorescent probe for dopamine sensing. It provided a sensitive detection platform with a limit of detection (LOD) lower than 17 nM [199]. 

### 4.5. g-C_3_N_4_-Based Electrochemiluminescent (ECL) Biosensors

Over the past several decades, many studies on electrochemiluminescence (ECL) biosensors have been conducted in various fields, such as chemical analysis and clinical diagnostics or food analysis. Electrochemiluminescence, or electrochemical chemiluminescence, is the light emission produced from molecular types by an electron transfer process. Additionally, ECL is triggered by an electrochemical reaction of the luminophores on an electrode surface. Moreover, the significant advantages of ECL are its high sensitivity and selectivity. In ECL biosensors, electrochemically generated intermediates endure an extremely exergonic reaction to turn out into an electronically excited state. ECL-based biosensors utilize specific biological diagnosis elements, such as enzymes, antibodies, aptamers, peptides, and proteins to selectively recognize a particular analyte and generate an ECL signal [208]. The basis of the method is on diagnosis interaction among biological cognizance elements and the corresponding targets by ECL release alterations. Accordingly, two main components are needed in standard ECL detection: ECL active types and biological cognizance elements.

Depending on the reaction that induces the ECL signal emission, there are several sensing systems for medical applications. 

In systems that are based on the chemical reactions of the luminophores and co-reactants, the chemical reaction between the luminophore and the co-reactant and is used for detecting diverse biomarkers.

The second type is systems that involve the co-reaction accelerator-involved reactions. In these systems, the reaction mixture is mixed with co-reaction accelerators. These accelerators are involved in generating electrochemiluminescent reactions in terms of facilitating the ECL reaction rate of co-reactant to produce several intermediates. 

In systems that incorporate resonance energy transfer (RET) reactions, instead of using only one luminophore, the signal is emitted via two different emitters by incorporating a RET. 

**Figure 4:**
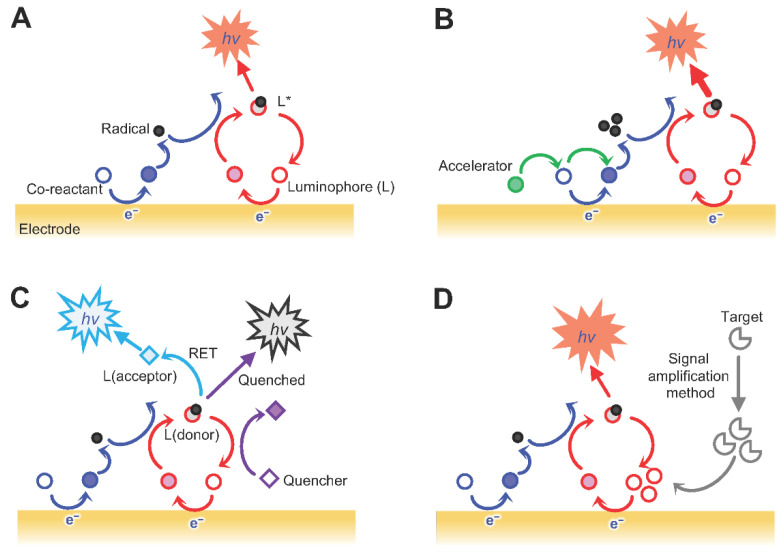
Differet categories of ECL systems. (**A**) Luminophore and co-reactant-involved reaction-based system; (**B**) co-reaction accelerator-involved reaction-mediated system; (**C**) resonance energy transfer (RET) reactions-incorporated system; and a (**D**) signal amplification method-incorporated system. Adapted from Ref. [209] under the terms and conditions of the Creative Commons Attribution (CC BY) license.

For systems that incorporate an enzyme reaction-based signal amplification, binding events between target analytes and probe DNAs initiate. High sensitivity and extension of the dynamic range of the modulation are some of the benefits of these systems [209]. Figure 4 represents the metioned types of ECL biosensors based on the reactions leading to ECL signal emission. 

g-C_3_N_4_ has a large surface area, and this carbon-based material can enable more sites to sequester charge carriers. Additionally, g-C_3_N_4_ has high electron conductivity, and they can successfully separate and then transfer charge carriers [208]. Some of the electrochemiluminescent biosensors are represented in Table 4.

Wu et al. developed an ECL immunosensor to detect the cancer biomarker CA125; nevertheless, its relatively low concentration in human body fluids limits the conventional methods. The disposable and label-free biosensor provided a sensitive detection via ECL emission when multifunctional g-C_3_N_4_ captures the CA125 tumor marker in the range from 0.001 to 5 U/mL, with a LOD of 0.4 mU/mL [213]. Wang et al. proposed a novel ECL bioassay system for detecting the HL-60 cancer cells based on g-C_3_N_4_ nanosheets and Ag–PAMAM–luminol nanocomposites (Ag–PAMAM–luminol NCs), where g-C_3_N_4_ nanosheets were applied as a reductive–oxidative ECL emitter. The overlapping of the ECL spectrum of g-C_3_N_4_ nanosheets and the adsorption spectrum of Ag nanoparticles as well as luminol oxidative–reductive ECL emissions simultaneously contributing to the sensitive detection of the HL-60 cancer cells, with 150 cells as the limit of detection [222]. 

## 5. Conclusions and Future Perspectives

The early diagnosis of diseases is the best way to improve the treatment prognosis and decrease the side effects of illnesses. Biosensors based on nanomaterials are efficient for this approach due to the high and rapid sensitivity in diagnosing the target molecules that arises from the specific properties of nanomaterials. In recent years, the nanosheets of g-C_3_N_4_ and their derivatives have attracted a lot of interest owed to their outstanding optical properties (high photoluminescence yield), high surface area, electrical conductivity, antimicrobial activity, and good thermal and chemical stability. Several simple and high-yield methods have been used to synthesize g-C_3_N_4_-based materials, such as the pyrolysis of low-cost materials, including melamine and urea. C_3_N_4_-based materials have also been used in various biosensors (SPR, EC, PCL), which demonstrates that they are promising candidates in this field. Moreover, g-C_3_N_4_-based biosensors show high and rapid sensitivity for detecting diseases, such as cancer; other targets in biological samples; or even the detection of pollutants. Thus, g-C_3_N_4_ is a new carbon-based 2D nanomaterial for biosensing, and it is expected that in the near future, g-C_3_N_4_-based biosensors will be improved in order to be more sensitive in diagnosis and functionalized in order to have more selectivity to attach the receptors. We anticipate that further research will be conducted on addressing the intrinsic shortcomings attributed to g-C_3_N_4_, including poor specific surface area, limited light absorption range, and poor dispersibility in organic and aqueous media.

## Figures and Tables

**Table 1:** Comparison of different biomarkers detection using electrochemical techniques.

Method	Interface	Biomarker	LOD	Dynamic Range	Ref.
Electrochemistry	IL-CNNS	2,4-Dichlorophenol	0.0062 μM	0.02–160 μM	[87]
Electrochemistry	Cu-Al_2_O_3_-g-C_3_N_4_-Pd	amyloid β-protein	3.3 fg/mL	10 fg/mL–100 ng/mL	[88]
Electrochemistry	CeO_2_/g-C_3_N_4_	anti-depressant drug Agomelatine (AG)	0.96 ng/mL	1–20 ng/mL	[89]
Electrochemistry	PEDOT/h-CN	ascorbic acid (AA) acetaminophen (AP)	1.51 μM 0.49 μM	4–20, 20–1800 μM 1–10, 10–50 μM	[90]
Electrochemistry	MoS_2_QDs@g-C_3_N_4_@CS-AuNPs	PSA	0.71 pg/mL	-	[40]
Electrochemistry	mpg-C_3_N_4_	Avian Leukosis Viruses	120 TCID_50_/mL	-	[91]
Electrochemistry	MIP/g-C_3_N_4_/FTO	bisphenol A	23 μmol L^−1^	5–200 μmol L^−1^	[92]
Electrochemistry	Ag/g-C_3_N_4_	CA 19-9	1.2 mU mL^−1^	5.0 mU mL^−1^–50 U mL^−1^	[93]
Electrochemistry	Au/ g-C_3_N_4_	chronic lymphocytic leukemia	20 pM	0.6 nM–6.4 nM	[94]
Electrochemistry	Au/mpg-C_3_N_4_	Cr(VI)	14 ppb	100–1000 ppb	[95]
Electrochemistry	g-C_3_N_4_/GO	pesticide	8.3 nM	0.045–213 μM	[96]
Electrochemistry	g-C_3_N_4_-E-PEDOT	acetaminophen	0.034 μM	0.01–2.0, 2.0–100 μM	[97]
diasadiElectrochemistry	C-g-C_3_N_4_	diphenylamine	0.009 μM	0.008–682 μM	[98]
Electrochemistry	g-C_3_N_4_/CuO	dopamine	1 × 10^−10^ mol L^−1^	2 × 10^−9^–7.11 × 10^−5^ mol L^−1^	[99]
Electrochemistry	Ru^0^ /PANI@g-C_3_N_4_	Bisphenol-A	0.18 nM	0.01–1.1 μM	[100]
Electrochemistry	Co_3_O_4_/g-C_3_N_4_	environmental phenolic hormones	3.3 × 10^−9^ mol L^−1^	1.0 × 10^−8^–1.2 × 10^−5^ mol L^−1^	[101]
Electrochemistry	V_2_O_5_/g-C_3_N_4_/PVA	folic acid	0.0017 μM	0.01–60 μM	[102]
Electrochemistry	VC/g-CN NSs	Furazolidone	0.5 nM	0.004−141 μM	[103]
Electrochemistry	g-C_3_N_4_/MoO_3_	Furazolidone	1.4 nM	0.01–228 μM	[104]
Electrochemistry	g-C_3_N_4_@Au NPs	galectin-3	25.0 fg mL^−1^	0.0001–20.0 ng mL^−1^	[105]
Electrochemistry	Pt^2+^@g-C_3_N_4_	glucose	10 μM	13–2000 μM	[106]
Electrochemistry	g-C_3_N_4_	glucose	5 μM	50 μM–2 mM	[107]
Electrochemistry	g-C_3_N_4_/Fe_2_O_3_-Cu	glucose	0.3 μM	0.6 μM-2.0 mM	[108]
Electrochemistry	g-C_3_N_4_−CH	Hg(II)	0.010 μmol L^−1^	1.00−80.0, μmol L^−1^ 0.100−5.00 μmol L^−1^	[109]
Electrochemistry	g-C_3_N_4_ and Hg(II)-imprinted polymer	Hg(II)	0.018 nmol L^−1^	0.06–25 nmol L^−1^	[110]
Electrochemistry	Pt /g-C_3_N_4_/ Polythiophene	Hg^2+^	0.009 nM	1–500 nM	[111]
Electrochemistry	Utg-C_3_N_4_	Hg(II)	0.023 µg/L	0.1–15.0 µg/L	[112]
Electrochemistry	g-C_3_N_4_-F127-Au NSs	HSP90	2.67 µg/mL	3.5 µg/mL–2.43 mg/mL	[113]
Electrochemistry	Co_3_O_4_/g-C_3_N_4_	hydrazine	1 µM	5–1000 µM	[114]
Electrochemistry	S-g-C_3_N_4_/FTO	hydrazine	0.06 µM	60 µM–475 µM	[115]
Electrochemistry	PANI/g-C_3_N_4_/AgNPs	hydrazine	300 μM	5–300 mM	[116]
Electrochemistry	Cu/MnO_2_/g-C_3_N_4_	hydrogen peroxide	0.85 µM	10–20,000, 20,000–400,000 µM	[117]
Electrochemistry	Na,O-g-C_3_N_4_	hydrogen peroxide	0.05 µM	1 µM–50 µM	[118]
Electrochemistry	g-C_3_N_4_/HOPG	hydrogen peroxide	0.12 μM	0.12–120 μM	[119]
Electrochemistry	rGO/g-C_3_N_4_	Pb(II)	1.07 × 10^−12^ mol/L	-	[120]
Electrochemistry	CsTi_2_NbO_7_@g-C_3_N_4_	nitrite	2.63 × 10^−5^ mol/L	0.0999–3.15 mmol/L	[121]
Electrochemistry	ZSO-gCN	nitrobenzene	2.2 μM	30–100 μM	[122]
Electrochemistry	Ox-g-C_3_N_4_	Norovirus-Specific DNA	100 fM	-	[123]
Electrochemistry	g-CNNS	ochratoxin A	0.073 nM	-	[124]
Electrochemistry	AChE/CS/Pd WLNCs/g-C_3_N_4_	acetylthiocholine (ATCl)	0.67 nM	0.002–2.46 μM	[125]
Electrochemistry	g-C_3_N_4_	oxalic acid	0.75 × 10^−6^ mol L^−1^	(1–1000) × 10^−6^ mol L^−1^	[126]
Electrochemistry	g-C_3_N_4_/PEDOT-MeSH	paracetamol	1 μM	0.4–1280 μM	[127]
Electrochemistry	g-C_3_N_4_ /CuO	p-nonylphenol	1.2 × 10^−8^ mol·L^−1^	3.0 × 10^−8^–5.1 × 10^−6^ mol·L^−1^	[128]
Electrochemistry	HP5@AuNPs@g-C_3_N_4_	PSA	0.12 pg mL^−1^	0.0005–10.00 ng mL^−1^	[129]
Electrochemistry	AuNP/g-C_3_N_4_	PSA	5.2 pg mL^−1^	0.01–30 ng mL^−1^	[130]
Electrochemistry	g-C_3_N_4_/NiO	quercetin	0.002 μM	0.010–230 μM	[131]
Electrochemistry	Pt/g-C_3_N_4_/Polyaniline	Hg^2+^	0.014 nM	1–500 nM	[132]
Electrochemistry	Bi_2_Te_3_@g-C_3_N_4_ BNs	ractopamine (RAC)	1.77 nM	0.015–456.4 μM	[133]
Electrochemistry	AuOct-PEI-C_3_N_4_	sulfamethazine	6.9 × 10^−5^ ng·mL^−1^	0.0001–100 ng·mL^−1^	[134]

**Table 2:** Using photoelectrochemical (PEC) techniques for biomarkers detection.

Method	Interface	Biomarker	LOD	Dynamic Range	Ref.
PEC	ZnO@CdTe nanocable arrays/carboxylated g-C_3_N_4_	Proprotein convertase subtilisin/kexin type 6 (PCSK6)	2 pg/mL	10 pg/mL–20.0 ng/mL	[143]
PEC	ZnO/MoS_2_/g-C_3_N_4_	5-hydroxymethylcytosine (5hmC)	2.6 pM	0.01–200 nM	[144]
PEC	CuO-g-C_3_N_4_	aflatoxin B1	6.8 pg mL^−1^	0.01 ng mL^−1^–1 μg mL^−1^	[145]
PEC	TiO_2_/g-C_3_N_4_	alkaline phosphatase	0.03 U/L	-	[146]
PEC	g-C_3_N_4_	chloramphenicol	0.22 pM	1 pM–100 nM	[147]
PEC	g-C_3_N_4_/TiO_2_	ascorbic acid alkaline phosphatase	0.3 nM 0.1 mU/L	1 nM–10 μM 0.3 mU/L–1 U/L	[148]
PEC	AuNPs/g-C_3_N_4_	avian viruses	85 TCID_50_/mL	-	[149]
PEC	Zn _0.1_ Cd _0.9_S/g-C_3_N_4_	Carcinoembryonic Antigen	1.4 pg·mL^−1^	0.005 ng·mL^−1^–20 ng·mL^−1^	[150]
PEC	g-C_3_N_4_/CuInS_2_	Carcinoembryonic Antigen	5.2 pg mL^−1^	0.02−40 ng mL^−1^	[151]
PEC	g-C_3_N_4_/CdSe	Carcinoembryonic Antigen	0.21 ng mL^−1^	10 ng mL^−1^–100 µg mL^−1^	[152]
PEC	ZnO NDs@g-C_3_N_4_ QDs	CCRF-CEM cell	20 cell/mL	20–20,000 cell/mL	[153]
PEC	Ag_2_CrO_4_/g-C_3_N_4_/GO	chloramphenicol	0.29 pM	0.5 pM–50 nM	[154]
PEC	P-g-C_3_N_4_-WS_2_	5- formylcytosine	3.8 pM	0.01–200 nM	[155]
PEC	g-C_3_N_4_/Ti_3_C_2_	ciprofloxacin	0.13 nM	0.4–1000 nM	[156]
PEC	Cu-BTC MOF/g-C_3_N_4_	glyphosate	1.3 × 10^−13^ mol L^−1^	1.0 × 10^−12^–1.0 × 10^−8^ mol L^−1^ and 1.0 × 10^−8^–1.0× 10^−3^ mol L^−1^	[157]
PEC	g-C_3_N_4_@CdS QDs	Hg^2+^	12 nM	20–550 nM	[158]
PEC	TiO_2_/g-C_3_N_4_/ graphene	dopamine	0.02 μM	0.1 to 50 μM	[159]
PEC	GOx|g-C_3_N_4_-TiO_2_|ITO	glucose oxidase	0.01 mM	0.05–16 mM	[160]
PEC	GOx-β-Gal@Au NPs-g-C_3_N_4_- MnO_2_-TiO_2_/ITO	Glucose and Lactose	0.23 mM	0.008–2.50 mM	[161]
PEC	g-C_3_N_4_/ZnIn_2_S_4_	glucose	0.28 μM	1–10,000 μM	[162]
PEC	utg-C_3_N_4_/WO_3_/ITO	glucose	0.0001 mM	0.01–7.12 mM	[163]
PEC	Mn_3_(BTC)_2_/g-C_3_N_4_/TiO_2_	H_2_O_2_	0.001 μM	0.003–10 μM	[164]
PEC	g-C_3_N_4_/P3HT	H_2_O_2_	0.38 μM	1.0–800 μM	[165]
PEC	g-C_3_N_4_/CdS quantum dots	methylated RNA	3.53 pM	0.01-10 nM	[166]
PEC	g-C_3_N_4_/CdS quantum dots	DNA MTase	0.316 U/mL	1–80 U/mL	[167]
PEC	cg-C_3_N_4_	Metronidazole	0.005 µM	0.01–100 µM	[168]
PEC	Au/CeO_2_/g-C_3_N_4_	Microcystin-LR	0.01 pM	0.05–10^5^ pM	[169]
PEC	MoS_2_/g-C_3_N_4_/black TiO_2_	microRNA	0.13 fM	0.5 fM–5000 fM	[170]
PEC	CdS@g-C_3_N_4_	MicroRNA	0.05 fM	0.1 fM–1.0 nM	[171]
PEC	g-C_3_N_4_-MoS_2_@CdS:Mn	myoglobin	0.42 pg mL^−1^	1.0 pg mL^−1^–50 ng mL^−1^	[172]
PEC	PPy/g-C_3_N_4_/WO_3_ IOPCs	Oxytetracycline (OTC(	0.004 nM	0.01–5 nM	[173]
PEC	g-C_3_N_4_/WO_3_ IOPCs	Oxytetracycline (OTC(	0.12 nM	1 nM–230 nM	[174]

**Table 3:** Fluorescent techniques developed for various biomarkers.

Method	Interface	Biomarker	LOD	Dynamic Range	Ref.
Fluorescent	S-Doped g-C_3_N_4_ Pinhole Porous Nanosheets	Ag^+^	57 nM	0 to 1000 nM	[190]
Fluorescent	g-C_3_N_4_	ascorbic acid	5.3nM	0–26.67 nM	[191]
Fluorescent	mpg-C_3_N_4_	Au^3+^	1.1 μM	-	[192]
Fluorescent	g-C_3_N_4_	chromium (VI)	0.15 μM	0.6 μM–300 μM	[193]
Fluorescent	g-C_3_N_4_	CN^−^ Cr_2_O_7_ ^2−^	1.5 µM 18 nM	- -	[194]
Fluorescent	g-C_3_N_4_	copper(II)	8 pM	0.01–0.4 nM	[195]
Fluorescent	g-C_3_N_4_	cytochrome C	2.6 nM	16–140 nM	[196]
Fluorescent	g-C_3_N_4_	Ag^+^ S^2^^−^	4.2 nM 3.5 nM	0–40 nmol /L 0–30 nmol/L	[197]
Fluorescent	g-C_3_N_4_ nanosheets/chromogenic	glutathione	0.01 μM	0.05 M L^−1^–1.0 M L^−1^	[198]
Fluorescent	g-C_3_N_4_	dopamine	0.017 μM	0–20 μM	[199]
Fluorescent	WS-g-C_3_N_4_@AuNCs	Fe^2+^ Cu^2+^	1.73 nmol L^−1^ 3.63 nmol L^−1^	-	[200]
Fluorescent	Fe-g-CNO	Fluoride Ions	1 × 10^−6^ M	-	[201]
Fluorescent	g-C_3_N_4_@CuMOFs	glucose	59 nM	0.1–22 μM	[202]
Fluorescent	g-C_3_N_4_−MnO_2_	Glutathione	0.2 μM	-	[203]
Fluorescent	g-C_3_N_4_	Hemin	0.15 μM	0.5–25 μM	[204]
Fluorescent	g-C_3_N_4_	H_2_O_2_	0.07 μM	0.1–100 μM	[205]
Fluorescent	g-C_3_N_4_–Dopa	laccase activity	2 U L^−1^	0–430 U L^−1^	[206]
Fluorescent	g-C_3_N_4_	metronidazole	0.008 μg ml^−1^	0.01–0.10 μg ml^−1^	[62]
Fluorescent	Fe_3_O_4_/g-C_3_N_4_/HKUST-1	ochratoxin A	2.57 ng/mL	5.0–160.0 ng/mL	[207]

**Table 4:** (ECL) methods for different biomarkers.

Method	Interface	Biomarker	LOD	Dynamic Range	Ref.
ECL	Au-g-C_3_N_4_ NHs	alpha fetoprotein	0.0005 ng mL^−1^	0.001–5 ng mL^−1^	[210]
ECL	g-C_3_N_4_	amyloid β peptides	3.25 fM	10 fM–0.1 μM	[211]
ECL	g-C_3_N_4_@Au NPs coated Pd NPs@NH_2_-MIL-53	amyloid β peptides	3.4 fg·mL^−1^	10 fg·mL^−1^–50 ng·mL^−1^	[212]
ECL	Fe_3_O_4_@g-C_3_N_4_	CA125	0.4 mU·mL^−1^	0.001–5 U·mL^−1^	[213]
ECL	Ag-doped g-C_3_N_4_	concanavalin A	0.0003 ng·mL^−1^	0.001–50 ng·mL^−1^	[214]
ECL	g-C_3_N_4_	tyramine	1.79 nmol L^−1^	1 × 10^−8^ −1 × 10^−3^ mol L^−1^	[215]
ECL	C-g-C_3_N_4_/CuO	dopamine	8.2 nM	10 nM–1 mM	[216]
ECL	g-C_3_N_4_ NSs–PTCA	dopamine	2.4 pM	6.0 pM–30.0 nM	[217]
ECL	AuNF@g-C_3_N_4_–PAN	dopamine	1.7 × 10^−9^ M	5.0 × 10^−9^–1.6 × 10^−6^ M	[218]
ECL	g-C_3_N_4_ NSs-rGO/S_2_O_8_ ^2−^	folic acid	62 pM	0.1–90 nM	[219]
ECL	ZnO@g-C_3_N_4_	fipronil	1.5 nmol L^−1^	5–1000 nmol L^−1^	[220]
ECL	Au-g-C_3_N_4_	Nuclear factor-kappa B	5.8 pM	-	[221]
ECL	g-C_3_N_4_ nanosheets and Ag-PAMAM-luminol	HL-60 cancer cells	150 cells	200–9000 cells·mL^−1^	[222]
ECL	C- g-C_3_N_4_	insulin	33 fg·mL^−1^	0.1 pg·mL^−1^–20.0 ng·mL^−1^	[223]
ECL	C_60_/g-C_3_N_4_ NS	melamine	1.3 × 10^−13^ M	2.7 × 10^−11^–1.9 × 10^−8^ M	[188]
ECL	g-C_3_N_4_/K_2_S_2_O_8_	methotrexate (MTX)	0.27 pM	1 pM–10 μM	[224]
ECL	g-C_3_N_4_@AuNPs	miRNAs	0.3 fM	1 fM–10 pM	[225]
ECL	Ce-MOF@g-C_3_N_4_/Au	N-terminal pro-B-type natriuretic peptide	3.59 pg mL^−1^	0.005–20 ng mL^−1^	[226]
ECL	g-C_3_N_4_ NSs	Pyrophosphate Ion	75 pM	2.0–800 nM	[227]
ECL	AuNPs/g-C_3_N_4_	squamous cell carcinoma antigen (SCCA)	0.4 pg·mL^−1^	0.001–10 ng·mL^−1^	[228]
ECL	Lum-AuNPs@g-C_3_N_4_	tumor exosomes	39 particles μL^−1^	-	[229]
ECL	g-C_3_N_4_ NS/TEA/Cu@Cu_2_O	microRNA-21	48 aM	-	[230]
ECL	g-C_3_N_4_/PDDA/CdSe	VEGF_165_	0.68 pg mL^−1^	2 pg mL^−1^–2 ng mL^−1^	[231]

## Data Availability

Not available.

## Abbreviations

**CV**	Cyclic Voltammetry
**EC**	Electrochemical Biosensor
**ECL**	Electrochemiluminescent Biosensor
**FCS**	Fluorescence Correlation Spectroscopy
**FI**	Fluorescence Intensity
**FLIM**	Fluorescence Lifetime Imaging
**FRET**	Forster Resonance Energy Transfer
**g-C_3_N_4_**	Graphite Phase Carbon Nitride
**PCL**	Photochemoluminescence Biosensor
**SPR**	Surface Plasmon Resonance
**SPW**	Surface Plasmon Wave
